# Correction to “Neoadjuvant ceritinib treatment in *ALK* ‐rearranged locally advanced adenosquamous carcinoma: A case report”

**DOI:** 10.1111/1759-7714.15048

**Published:** 2023-07-30

Mai S, Wang Y, Wang X, Yang W, Gao H, Xu Z, et al. Neoadjuvant ceritinib treatment in *ALK*‐rearranged locally advanced adenosquamous carcinoma: A case report. Thorac Cancer. 2022;13(15):2275–78. https://doi.org/10.1111/1759-7714.14558

There was a mistake in the labeling of P63 and P40 in Figure 2a. The revised figure and caption are shown below:

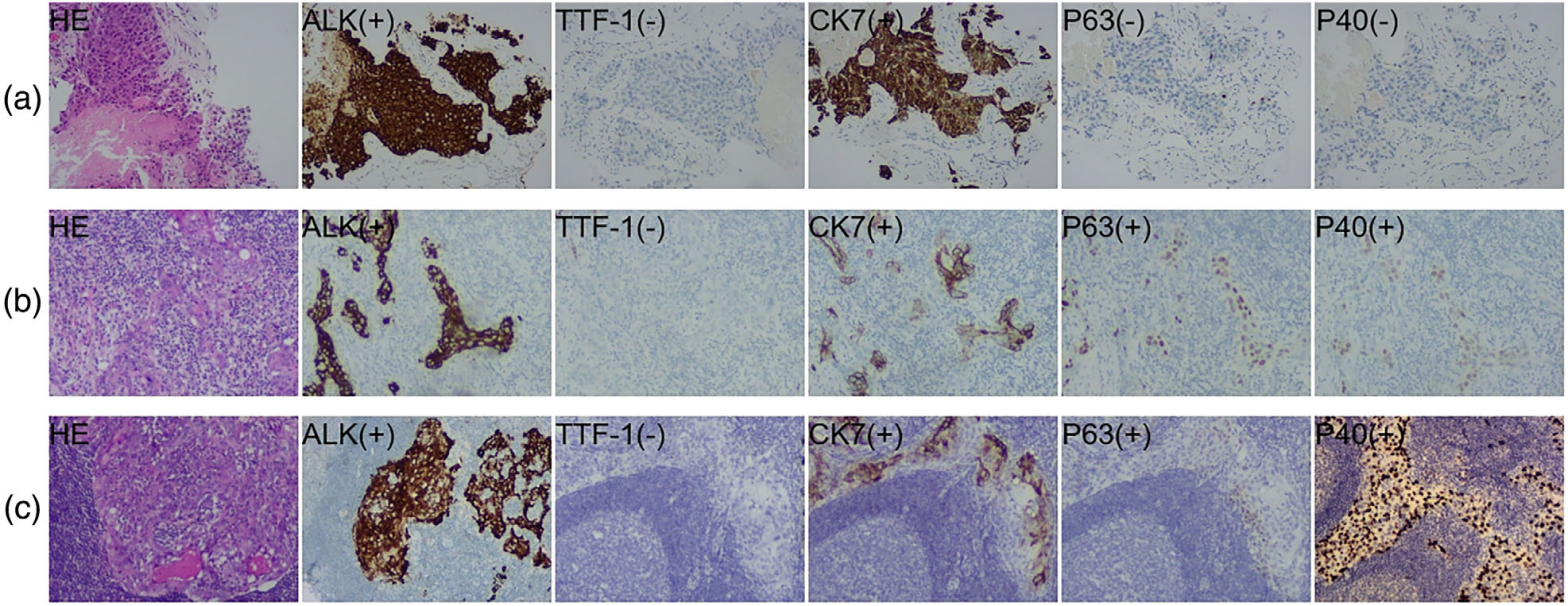

**FIGURE 2** Hematoxylin–eosin (HE) staining and immunohistochemistry (IHC) images (100× magnification). (a) The HE staining of samples punctured before neoadjuvant ceritinib shows nests of tumor cells with necrosis. The IHC examinations reveal positive ALK (D5F3), CK7, and negative TTF‐1, P63, P40, supporting the diagnosis of ADC. (b) In the surgically‐resected specimen after neoadjuvant ceritinib, there is a significant decreased amount of tumor cells in the tumor bed, most of the interstitial fibrosis, and more lymphocytes are scattered, where round tumor cell nests with eosinophilic cytoplasm and vesicular nuclei are seen. The positive IHC of ALK (D5F3), CK7, P63, and P40 indicated the histology of ASC. (c) The right upper paratracheal lymph node, surgically‐resected after neoadjuvant ceritinib, shows scattered nests of tumor cells with eosinophilic cytoplasm and vesicular nuclei. The positive IHC of ALK (D5F3), CK7, P63, and P40 indicated the histology of ASC.

We apologize for these errors.

